# 

**Published:** 1899-03-04

**Authors:** 


**Notices of Births, Deaths, and Marriages** are inserted in this column at a charge of 2s. 6d. for an announcement not exceeding four lines.

### NOTICE TO CORRESPONDENTS.

All MS., Letters, Books for Review, and other matters intended for the Editor should be addressed The Editor, "The Hospital," The Hospital Building, 28 & 29, Southampton Street, Strand, W.C.

The Editor cannot undertake to return rejected MS., even when accompanied by stamped directed envelope.

**Notice to Advertisers and Subscribers.**

All Advertisements, Orders for Copies of The Hospital, and Business Communications should be addressed to **THE MANAGER (Not to the Editor),** The Hospital, The Hospital Building, 28 & 29, Southampton Street, Strand, London, W.C.

The Cost of Subscription, post free, is as follows:—Per week, 2½d.; per quarter, 2s. 8d.; per half-year, 5s. 3d.; per annum, 10s. 6d. Foreign:—Per annum, 15s. 2d., payable, in all cases, in advance.

**NOTICE.—Vol. XXIV. of "The Hospital" (April, 1898, to September, 1898), in handsome cloth binding, is now on sale at the Office of this Journal, price 7s. 6d. Cases for binding the Numbers contained in this Volume 1s. 6d. Index to Vol. XXIV., 2½d., post free.**

**The Monthly Part for March is now ready. Price 6d., by post 8d.**

### BOOKS RECEIVED.

The Scientific Press.

"The London Water Supply: A Retrospect and a Survey." By Richard Sisley, M.D., Lond., M.D. (State Med.)

John Wright and Co.

"Physician's Book for Private Formulæ; with Posological Table."

James Bowden.

"The Secret of Good Health and Long Life." By Haydn Brown L.R.C.P., L.R.C.S.Edin.

Edward Lloyd.
"The Municipal Year Book for 1899." Edited by Robert Donald.

University Correspondence College Press.

"The Calendar, 1898-9," with "The London Matriculation Directory, 1899."

J. Whitaker and Sons.
"Whitaker's Naval and Military Directory and Indian Army List, 1899."

Oliver and Boyd.
"Notes on Surgery for Nurses." By Joseph Bell, M.D., F.R.C.S., Ed.

The Ohio Valley Co., Cincinnati.
"King's American Dispensatory." Vol. I.

Adam and Charles Black.
"The Englishwoman's Year Book and Directory, 1899." Edited by Emily Janes.

T. B. Browne.
"The Advertiser's A B C."

